# Effects of Cycling Intensity on Acute Signaling Adaptations to 8-weeks Concurrent Training in Trained Cyclists

**DOI:** 10.3389/fphys.2022.852595

**Published:** 2022-03-23

**Authors:** T. W. Jones, L. Eddens, J. Kupusarevic, D. C. M. Simoes, M. J. W. Furber, K. A. Van Someren, G. Howatson

**Affiliations:** ^1^ Department of Sport Exercise and Rehabilitation, Northumbria University, Newcastle-upon-Tyne, United Kingdom; ^2^ Population Health Sciences Institute, Newcastle University, Newcastle-upon-Tyne, United Kingdom; ^3^ Department of Psychology and Sports Science, University of Hertfordshire, Hatfield, United Kingdom; ^4^ Sports Lab North West, Letterkenny Institute of Technology, County Donegal, Ireland; ^5^ Water Research Group, North West University, Potchefstroom, South Africa

**Keywords:** interference effect, anabolic, combined exercise, strength, endurance

## Abstract

This study examined whether the intensity of endurance stimuli modifies the adaptation in strength and endurance following concurrent training and whether the acute molecular response to concurrent exercise is affected by training status. Using a parallel group design, trained cyclists were randomized to either resistance exercise followed by moderate intensity continuous training (RES + MICT, *n* = 6), or resistance exercise followed by work matched high intensity interval training (RES + HIIT, *n* = 7), across an 8 weeks training programme. A single RES + MICT or RES + HIIT exercise stimulus was completed 1 week before and within 5 days of completing the training programme, to assess phosphorylation of protein kinases of the mTOR and AMPK signaling pathways. There were no main effects of time or group on the phosphorylation of protein kinases in response to concurrent exercise stimulus pre- and post-training intervention (*p* > 0.05). Main effects of time were observed for all maximal strength exercises; back-squat, split-squat, and calf-raise (*p* < 0.001), with all improving post intervention. A time × group interaction was present for V̇O_2peak_, with the RES + MICT group displaying a preferential response to that of the RES + HIIT group (*p* = 0.010). No time nor group effects were observed for 5 min time trial performance, power at 2 and 4 mmol L^−1^ (*p* > 0.05). Whilst preliminary data due to limited sample size the intensity of endurance activity had no effect on performance outcomes, following concurrent training. Further, the acute molecular response to a concurrent exercise stimulus was comparable before and after the training intervention, suggesting that training status had no effect on the molecular responses assessed.

## 1 Introduction

A concurrent training model has long been associated with an interference effect, whereby strength adaptation is inhibited when practicing concurrent training *vs* strength training in isolation (Hickson 1980). Conversely, a recent meta-analysis has indicated that concurrent training does not compromise maximal strength nor hypertrophic development irrespective of training modality, frequency or an individual’s age, but can attenuate explosive strength development (Schumann et al., 2021). However, this meta-analysis was unable to assess the role of endurance training intensity, due to inconsistent reporting within the studies included. Furthermore, the participants were classified as either “untrained” or “active.” As such, the role of endurance training intensity within the concurrent training paradigm in an endurance trained cohort is yet to be fully elucidated.

Exercise intensity is a key training variable. High intensity interval training (HIIT) can offer adaptations consistent, if not superior to that of traditional endurance training, with regards to aerobic capacity (Wisløff et al., 2007; Hwang et al., 2011) and is effective in eliciting endurance adaptations in well-trained cohorts (Skovereng et al., 2018). If endurance activity is purported to be antagonistic to strength adaptation, it would seem logical that a greater endurance exercise intensity would exacerbate the issue. This could be particularly relevant given that AMPK phosphorylation is greater following higher intensity (85% V̇O_2peak_) *vs* lower intensity cycling exercise (35% V̇O_2peak_) in healthy males, supporting the intensity-dependent regulation of AMPK (Rose et al., 2009). It has been suggested that AMPK induced blunting of mTOR signaling and subsequent protein synthesis may be a contributing factor to the interference effect (Hamilton and Philp 2013). However, the relevance of AMPK activation status should be treated with caution, as there are data to suggest that AMPK phosphorylation does not inhibit acute growth-related responses after subsequent strength stimuli in moderately trained males (Apró et al., 2015). Furthermore, recent work has reported similar anabolic signaling responses following combined strength and high- and moderate intensity endurance exercise in endurance trained cyclists (Jones et al., 2021), and that interference characteristics may be avoided if high intensity interval type endurance training is implemented alongside strength training (Vechin et al., 2021).

Experimentally, just two groups have explored the question of endurance exercise intensity in the context of concurrent training, specific to recreationally active individuals (Silva et al., 2012; Fyfe et al., 2016a). Silva et al. (Silva et al., 2012) reported no interference effect, nor any group differences across strength and endurance outcomes following the manipulation of endurance exercise intensity. In contrast, Fyfe et al. (Fyfe et al., 2016a) did report an interference effect across lower-body strength and power measures, but similarly failed to observe any effect of endurance exercise intensity following a concurrent training intervention. The differences in the studies’ findings may be attributable to the differing populations employed, these being young women (Silva et al., 2012) and recreationally active males (Fyfe et al., 2016a). Furthermore, Silva et al. (Silva et al., 2012) employed strength training and interval running over 11 weeks and Fyfe et al. (Fyfe et al., 2016a) employed strength training and interval cycling over 8-weeks. Regardless of whether an interference effect does exist across a training period, it is likely to be of greater importance to the athlete to understand whether a lower or higher intensity endurance component might be advantageous to performance outcomes following concurrent training.

Research supports the inclusion of lower-body strength training for endurance cycling cohorts, with previous work reporting beneficial effects of strength training on cycling performance (Rønnestad et al., 2010; Rønnestad et al., 2011). Therefore, a trained endurance cohort should prove a suitable population to investigate the role of endurance exercise intensity within a concurrent training programme. The training status of the individual will likely have an important role on the adaptive response to a concurrent training intervention. Specifically, both endurance and strength training status might modify the early molecular signaling responses to exercise, with an attenuated response amongst trained phenotypes and a generic molecular footprint response in untrained cohorts (Coffey and Hawley 2017). Observing the early molecular response to a concurrent exercise stimulus pre- and post-training intervention might help to substantiate these suggestions.

The purpose of this study was twofold. Firstly, to observe whether the acute molecular response to concurrent exercise stimuli is differentially affected in relation to the endurance intensity prescribed throughout the training intervention. Secondly, to examine whether the intensity of endurance stimuli throughout a short-term concurrent training block affected performance outcomes in an endurance cycling trained cohort.

## 2 Materials and Methods

### 2.1 Design

**FIGURE 1:**
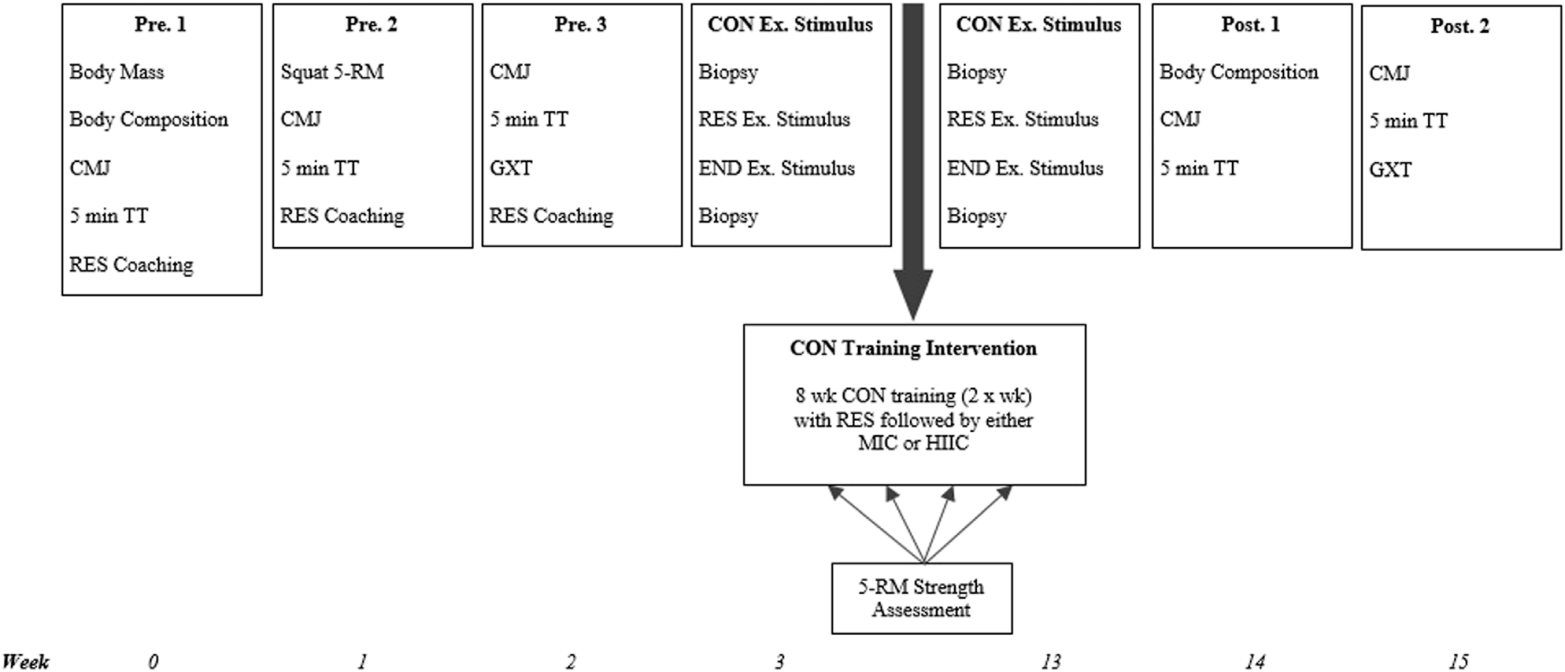
Study schematic. CMJ = counter-movement jump; TT = time trial; RES = resistance exercise; END = endurance exercise; CON = concurrent; Ex. = exercise; wk = week; MICT = moderate intensity cycling; HIIT = high intensity interval training; 5-RM = 5-repitition maximum; GXT = graded exercise test; MICT = moderate intensity cycling; HIIT = high intensity interval cycling.

The study utilized a repeated-measures, parallel group design. Following three preliminary trials for familiarization to procedures and collection of baseline data, participants were ranked on predicted 1-RM back-squat performance. Participants were subsequently randomized, in a stratified fashion, to either, 1) resistance exercise followed by moderate intensity continuous training (RES + MICT, *n* = 6), or 2) resistance exercise followed by work matched high intensity interval training (RES + HIIT, *n* = 7). Participants then completed an 8 weeks training programme, with two group-specific sessions performed per week, separated by ≥ 48 h between sessions. Maximal strength was assessed at 2 weeks intervals, while other performance outcomes were repeated post-intervention. A single group-specific exercise stimulus was completed at least 1 week before and within 5 days of completing the training programme, to assess phosphorylation of protein kinases associated with the mTOR and AMPK signaling pathways. A schematic of the experimental timeline is presented in Figure 1.

Preliminary visits were used to collect descriptive data; height and body mass, provide familiarization to and collect baseline data for performance outcomes; aerobic thresholds, back-squat five repetition maximum (5-RM), countermovement jump height (CMJ), 5 min time trial (TT), and body composition. The remainder of the preliminary visits were used to coach the lower-body strength exercises included in the training programme; split-squat and calf-raises. Peak oxygen uptake (V̇O_2peak_) and 5-RM data were used to prescribe relative exercise intensities for the single concurrent exercise stimulus and training intervention. The single concurrent exercise stimulus required participants to complete RES (6 × 8 back-squat repetitions at 80% predicted 1-RM) followed by either MICT (continuous 40 min cycling at 65% V̇O_2peak_) or HIIT (40 min cycling with 3 min intervals of 85 and 45% V̇O_2peak_). Muscle biopsies were collected at rest and 3 h post-RES. The same intra-session order i.e., resistance followed by endurance, was used throughout the training programme, with session load periodized across the 8 weeks duration (Table 1). The intra-session order used has been reported to be preferential for lower-body strength adaptation across a short-term concurrent training programme (Eddens et al., 2018).

**TABLE 1:** Details of the 8 weeks resistance training programme.

Phase	Week	Session	Detail
	1	1	5RM assessment
1	1	2	3 sets, 10 reps @ 75% 1RM
1	2	3	3 sets, 6 reps @ 85% 1RM
1	2	4	3 sets, 10 reps @ 75% 1RM
	3	5	5RM assessment
2	3	6	3 sets, 8 reps @ 80% 1RM
2	4	7	3 sets, 5 reps @ 87% 1RM
2	4	8	3 sets, 8 reps @ 80% 1RM
2	5	9	3 sets, 5 reps @ 87% 1RM
2	5	10	3 sets, 8 reps @ 80% 1RM
	6	11	5RM assessment
3	6	12	3 sets, 6 reps @ 85% 1RM
3	7	13	3 sets, 4 reps @ 90% 1RM
3	7	14	3 sets, 6 reps @ 85% 1RM
3	8	15	3 sets, 4 reps @ 90% 1RM
	8	16	5RM assessment

Note: 5RM, 5 repetition maximum; 1RM, 1 repetition maximum; reps = repetitions.

### 2.2 Participants

Fourteen men volunteered to take part in the study; however, one participant withdrew due to circumstances unrelated to the study. Thirteen participants (age 30 ± 6 years; height 179 ± 4 cm; body mass 71.8 ± 7.4 kg; V̇O_2peak_ 55.9 ± 7.0 ml kg^−1^ min^−1^; back-squat 1-RM 107.9 ± 31.2 kg) completed the study. All participants were trained endurance cyclists with 4 ± 3 years competitive cycling experience, were currently performing 4 ± 1 cycling training sessions·wk-1 and were regularly competing (at least a Category 3 British Cycling license holder or an estimated 16.1 km time trial of ≤23 min). Participants had no resistance training history for ≥6 months prior to enrolment. After being informed of the potential benefits and risks and completing a questionnaire to assess for eligibility and contraindications to the study, participants volunteered to take part in the research by providing written, informed consent. All documentation and procedures were approved by the institutional research ethics committee, in accordance with the Declaration of Helsinki. Eight of the thirteen cyclists who participated in the present study, also participated in an acute repeated measures cross over study investigating the acute effects of the intensity of endurance stimuli on the phosphorylation of signaling proteins associated with the mTOR and AMPK networks (Jones et al., 2021). Within said acute study all participants completed three independent and different single exercise sessions on separate occasions, with no longitudinal intervention nor pre- and post-intervention assessments. Unlike the present study, which involves parallel groups, both completing two independent 8 weeks training interventions with assessments of signaling responses and performance outcomes conducted pre- and post-intervention.

### 2.3 Preliminary Testing

Preliminary visits were undertaken at least 1 week prior to the single concurrent exercise stimulus. At visit 1, data were collected for height and body mass (Seca 704 r, Seca., Hamburg, Germany), followed by an assessment of body composition. Maximal strength was assessed at visit 2, while data were collected for CMJ, aerobic profile and 5 min TT at visit 3, in all cases TT assessments were conducted after a recovery period of 60 min following aerobic profiles assessments. These preliminary visits were also used to familiarize participants with the CMJ and 5 min TT performance tests, in addition to coaching of the lower-body strength exercises to the strength-trained naïve cohort.

### 2.4 Assessment of Peak Oxygen Uptake

Detailed information on the protocols employed here is presented in Jones et al. (Jones et al., 2021). Briefly, an incremental lactate threshold (LT) assessment was conducted prior to the V̇O_2peak_ test, with the starting intensity selected (range: 125–200 W) with subsequent increases in the work rate of 25 W every 4 min. This assessment was terminated with a blood lactate concentration of ≥4 mmol L^−1^ (range: 4 – 7 stages). After completion of the lactate threshold assessment, a 15 min period of rest was initiated. Participants then cycled at a power output of 200 W using an electro-magnetically braked cycle ergometer (Velotron, RacerMate Inc., Seattle, United States). Power output was subsequently increased by 4 W every 10 s (24 W min^−1^) until volitional exhaustion.

### 2.5 Body Composition

Height (stretch stature), mass and skinfolds were collected in accordance with the standard procedures recommended by the International Society for the Advancement of Kinanthropometry; ISAK (Marfell-Jones et al., 2006). Measures were recorded for eight skinfold thicknesses (triceps, subscapular, biceps, iliac crest, supraspinale, abdominal, anterior thigh, and medial calf), using Harpenden skinfold calipers (Baty International., West Sussex, United kingdom). Each site was measured in duplicate, with a third collected if the technical error of measurement (TEM) threshold advised by ISAK was breached for a given site. The equation adapted from (Withers et al., 1987) was used to estimate percent body fat (Eq. (1) *BF% = 495/*(1.0988–0.0004* Ʃ7)*-450*) and calf girth was also measured. This enabled measures for sum of 7 skinfolds (Ʃ7), body density, body fat percentage (BF%), fat mass, and fat-free mass. All assessments were conducted by the same certified anthropometrist, with a mean TEM of 1.95% across the respective measures.

### 2.6 Counter-Movement Jump

The CMJ protocol was always preceded by a standardized 5 min warm-up at an intensity of 50% V̇O_2peak_ on the same cycle ergometer detailed previously, followed by a 5 min standardized dynamic warm-up consisting of heel to toe walking, goblet squats, squat jumps, and stiff-leg jumps. Counter-movement jump (CMJ) performance was assessed using the OptoJump system (OptoJump, Microgate S. r.l., Bolzano, Italy), with three maximal efforts performed on each testing occasion, each separated by 60 s rest. Participants were instructed to place their hands on their hips, descend rapidly to ∼90° knee joint angle, and then jump as high as possible. Standardized verbal encouragement was provided for each effort and the peak value generated across the three repetitions was used for data analysis. The intra-individual reliability of this measure returned a coefficient of variation of 0.9%.

## 3 Five Minute Time Trial

Following a standardized 5 min warm-up at an intensity of 50% V̇O_2peak_, participants completed a 5 min TT on the same cycle ergometer detailed previously. The assessment required participants to maintain the highest power output possible over a 5 min period. The trial started with the ergometer set in the lowest possible gear ratio, whereby after a 3 s count-down, the participant was responsible for manipulating gearing to a desired level. Feedback of performance data was withheld, except time elapsed, which was communicated only at the halfway point (2.5 min) and participants were permitted to change gears as and when they felt necessary. Heart rate was continually recorded throughout each trial, using wireless telemetry (T31 transmitter, Polar Electro Ltd., Kempele, Finland) and participants were cooled with an electric fan on a standardized setting.

### 3.1 Maximal Strength Testing

Detailed information on the protocols employed here is presented in Jones et al. (Jones et al., 2021). Briefly, maximal strength was predicted from participants’ 5-RM performance in the three lower-body exercises; back-squat, split-squat, and calf-raise. Maximal strength was predicted from participants’ 5-RM performance in the relevant exercise, using the following; Eq. (2) 1-RM = 100 · rep wt/(48.8 + 53.8 · exp [-0.075 · reps] (Wathan 1994), which previously reported good agreement with 1-RM performance in individuals naïve to strength training (LeSuer et al., 1997). The three strength exercises used within this study were the back-squat, split-squat, and calf-raise. The squat technique is reported to provide a potent stimulus of the *vastus lateralis*, comparative to that of alternate lower-body strength exercises (Ebben et al., 2009). Further, these three exercises are reported to improve parameters of strength, jump height, and muscle CSA amongst trained cyclists (Rønnestad et al., 2010; Rønnestad et al., 2017). The assessments were conducted in line with standardized procedures (Ebben et al., 2009; Rønnestad et al., 2012) and if more than one exercise was being assessed, a back-squat, split-squat, calf-raise order was followed, with a 10-min rest period provided between exercises.

### 3.2 Single Concurrent Exercise Stimulus

#### 3.2.1 Exercise and Dietary Control

Detailed information on the protocols employed here is presented in Jones et al. (Jones et al., 2021). Briefly, for 24 h prior to an experimental trial, participants refrained from structured exercise and consumed a standardized diet. No participants reported performing any strenuous or “heavy” exercise for 72 h prior to the experimental trials. Dietary intake was controlled for 24 h prior to arrival at the laboratory, through to completion of the final visit. Daily dietary intake was standardized (6 g kg^−1^ d^−1^ carbohydrate, 1.3 g kg^−1^ d^−1^ protein, 0.98 g kg^−1^ d^−1^ fat), with the evening meal (7:00 p.m.) and breakfast meal (6:00 a.m.) prior to the visit standardized at 3 g kg^−1^ d^−1^ carbohydrate, 0.5 g kg^−1^ d^−1^ protein, 0.3 g kg^−1^ d^−1^ fat and 1 g kg^−1^ d^−1^ carbohydrate, 0.1 g kg^−1^ d^−1^ protein, <0.01 g kg^−1^ d^−1^ fat, respectively.

### 3.3 Resistance Exercise Stimulus

Detailed information on the protocols employed here is presented in Jones et al. (Jones et al., 2021). Briefly, participants completed two warm-up sets of the back-squat (10 and 8 repetitions at 40 and 60% of predicted 1-RM, respectively). Participants completed 6 × 8 repetitions at 80% of predicted 1-RM, with the rest period between each set standardized at 3 min. Participants commenced the endurance exercise stimulus (described subsequently) within 5 min of completing RES.

### 3.4 Endurance Exercise Stimulus

Detailed information on the protocols employed here is presented in Jones et al. (Jones et al., 2021) along with a schematic representation of the protocols. Briefly, participants completed either moderate intensity cycling (MICT) or work matched high intensity interval cycling (HIIT), dependent upon randomization. MICT entailed constant load cycling at power output at 65% V̇O_2peak_ for 40 min, while HIIT required participants to perform 3 min intervals of 85% (6 repetitions) and 45% (5 repetitions) V̇O_2peak_, using the cycle ergometer. The exercise of 3 min intervals of 85 and 45% V̇O_2peak_ provided a total mechanical work matched high intensity intervention (Seiler and Tønnessen 2009). Both protocols contained a warm-up and cool down and are presented in Jones et al. (Jones et al., 2021). Heart rate was recorded throughout each trial, while visual feedback of time elapsed, power output, and pedal cadence were made available to participants. Power output was controlled via the cycle ergometer and maintained at power output at the appropriate % of V̇O_2peak_ established during the incremental assessment of V̇O_2peak_. If the cyclist’s cadence decreased, resistance increased and *vice versa* to maintain the pre-set power output.

### 3.5 Muscle Tissue Sampling

A single RES + MICT or RES + HIIT exercise stimulus was completed 1 week before and within 5 days of completing the training programme, to assess phosphorylation of protein kinases of the mTOR and AMPK signaling pathways. Muscle tissue sampling was conducted prior to the RES + MICT or RES + HIIT exercise stimuli and 3 h post the cessation of exercise. Analyses quantified the phosphorylation of Akt, AMPKα2, ERK, HSP27, mTOR, p38α, p53, p70S6K and STAT2. Detailed information on the protocols employed here is presented in Jones et al. (Jones et al., 2021). Briefly, upon arrival at the laboratory (∼0,730 h), participants were screened for contraindications to the muscle biopsy procedure including bleeding diathesis or receiving anticoagulation, before resting in a supine position (10 min). Muscle samples were collected from the middle portion on the lateral aspect of the *vastus lateralis* muscle, using the micro-muscle biopsy technique. Samples were obtained under local anesthesia, with 2 ml of 1% lidocaine Hydrochloride (Hameln Pharmaceuticals., Gloucester, United kingdom) injected into the subcutaneous tissue of the biopsy site. After confirming that the anesthetic had taken affect (∼5 min), a 14-gauge co-axial needle was inserted ∼2 cm into the muscle (beyond the subcutaneous tissue). A disposable biopsy instrument (TSK Stericut Biopsy Needle 14 Gauge, TSK Laboratories, Tochigi, Japan) was subsequently inserted through the co-axial and discharged. A single muscle sample was collected (∼10–20 mg) and the tissue was immediately frozen in liquid nitrogen, before being stored at −80°C until subsequent analysis. If required, a second pass was completed, with the biopsy instrument rotated 180° inside the co-axial needle. Biopsies were obtained immediately prior to RES and 3 h after completion of RES, with participants resting in a waiting room for the interval between the end of exercise and the final biopsy. All within-trial biopsies were sampled from the same leg, while between-trial biopsies were sampled from alternate legs.

### 3.6 Training Intervention

Participants began the training intervention ≥1 week following the initial single concurrent exercise stimulus. The RES stimulus was identical between groups and was always completed first in the session, with MICT or HIIT commencing within 5 min of completing RES. The training intervention was modified to allow for an overload stimulus, by increasing load lifted following intermediary strength assessments, or by increasing the duration of the MIT or HIIT sessions. Participants were required to complete ≥95% of the scheduled training sessions, all of which were to be completed in the laboratory under supervision. A maximum of four participants could be trained in the laboratory at any one time, with the two investigators supervising and providing verbal encouragement to motivate participants to complete the sessions.

### 3.7 Resistance Training

The resistance training programme was performed twice per week and incorporated three strength exercises; the back-squat, split-squat, and calf-raise. Each visit started with the same standardized warm-up as completed prior to maximal strength testing, followed by two sets of back-squat of increasing load (40 and 60% of predicted 1-RM) and decreasing number of repetitions (10 and 8, respectively). A back-squat, split-squat, calf-raise order was followed and sets were separated by a 3 min rest period. Intermediary assessments of maximal strength were conducted throughout the intervention and session load was modified if maximal strength had increased. The resistance programme is presented in full in Table 1. The strength and conditioning coach cued the participants to complete the repetitions with maximal intended movement velocity (Behm 1996).

### 3.8 Endurance Training

Participants completed either MICT or work matched HIIT, within 5 min of completing the RES training stimulus. Session duration was modified at week five, to incorporate another set of intervals for the HIIT group, or another 6 min of cycling at power output at 65% V̇O_2peak_ for the MICT group. Participants were cooled with an electric fan on a standardized setting. Training and performance tests were performed on the same cycling ergometer. Power output was controlled via the cycle ergometer and maintained at power output at the appropriate % of V̇O_2peak_ established during the incremental assessment of V̇O_2peak_. If the cyclist’s cadence decreased, resistance increased and *vice versa* to maintain the pre-set power output.

### 3.9 Training Load Quantification

Laboratory (prescribed) and non-laboratory including any additional endurance training participants wished to perform (non-prescribed) training load was quantified for all endurance training completed by all participants. No additional strength training was permitted during the experimental period. Work performed (external load) during laboratory training visits was matched for the two groups, relative to maximal aerobic capacity. Heart rate, rate of perceived exertion (RPE), and session duration data were collected for both prescribed and non-prescribed endurance training performed across the intervention period. The internal training load was then quantified using the session RPE model (Foster et al., 2001) and by using duration in individual heart rate zones (Halson and Jeukendrup 2004), multiplied by the zone weighting factor i.e., 1, 2, 3, 4, or 5 to provide a training impulse (TRIMP) score expressed in arbitrary units (Halson 2014) and reflective of cardiovascular strain. RPE for the session was assessed with Borg’s modified CR-10 scale, with the score multiplied by session duration to provide total load also expressed in AU. These data were collected with the use of an online training survey sheet (www.docs.google.com), to assist the participants with logging the duration of the session and the associated RPE. This process was completed within 30 min of training session completion. Further, each participant was provided with a heart rate monitor (Polar A300 transmitter, Polar Electro Ltd., Kempele, Finland), with both laboratory and non-laboratory training session data to be uploaded to the manufacturer’s portal (www.flow.polar.com). A sync from the participant’s watch to the laboratory iPad was conducted at the end of each training session, which ensured that data from that laboratory session and any external training since the previous laboratory session, was uploaded to the manufacturer’s portal. The same device was used to monitor both laboratory and external heart rate responses.

### 3.10 Muscle Analysis

Detailed information on the protocols employed here is presented in Jones et al. (Jones et al., 2021) and the phosphorylation of the following targets were analyzed Akt, AMPKα2, ERK, HSP27, mTOR, p38α, p53, p70S6K and STAT2. Briefly, all muscle samples were analyzed using a human phospho-kinase array (Proteome Profiler; no. ARY003B, R&D Systems., Minneapolis, United States), as per the manufacturer’s instructions. Approximately 10 mg of muscle tissue was homogenized in ice-cold lysis buffer. Samples were rotated end-over-end for 30 min at 4°C and centrifuged at 13,000 g for 6 min, and the supernatant subsequently collected. Protein concentration was determined using a total protein assay (Pierce BCA Protein Assay; no. 23225, Thermo Scientific., Rockford, United States), with a starting range of 400 µg per array. The nitrocellulose membranes with spotted capture and control antibodies, were blocked with array buffer 1 for 1 h at room temperature on a rocking platform shaker. Cell lysates were then diluted to a final volume of 2 ml with array buffer 1 and membranes rocked in solution overnight at 4°C. Membranes were subsequently washed to remove unbound proteins and incubated for 2 h at room temperature with the respective antibody solution (diluted detection antibody cocktail A or B). After washing, membranes were incubated for 30 min in a diluted streptavidin horseradish-peroxidase solution and protected from light, while being rocked at room temperature. After being washed again, chemiluminescent detection reagents were spread evenly onto the membranes and incubated for 1 min, before removing excess solution and measuring the amount of bound phosphorylated protein with a 15 min exposure, using a Syngene G:Box XR5 imaging system with GeneSys analysis software (Syngene., Cambridge, United kingdom).

After imaging, the average signal produced at the duplicate capture spots was quantified for each phosphorylated kinase protein with the ImageJ application (National Institute of Health, United States). In brief, the region of interest on each membrane was measured with the same frame, producing a pixel density for each spot. An inverted value was calculated per protein, with net values calculated by subtracting the inverted background. Finally, a protein ratio value was calculated by taking a ratio of the net value over the reference control, allowing for the relative quantification of phosphorylation between experimental conditions.

### 3.11 Statistical Analysis

Data are presented as mean ± SD, with statistical significance set at *p* ≤ 0.05 a priori. Sphericity was assumed if Mauchly’s test score returned *p* ≥ 0.05, with Greenhouse-Geisser adjustments made where appropriate. All measures which were repeated at different time points throughout the training intervention i.e., maximal strength, were analyzed using a condition (RES + MICT *vs* RES + HIIT) by time-point (pre-vs post-intervention) repeated measures mixed model ANOVA. Further, single time point measures i.e., training load, were analyzed using an independent samples *t*-test (RES + MICT *vs* RES + HIIT). Significant main effects were further investigated using LSD post-hoc, pair-wise comparisons. All data analysis was performed using statistical software (IBM SPSS 22 for Windows., New York, United States). Due to the parallel group design and relatively low number of participants, where possible standardized effect size (Hedge’s *g*) analyses were used to interpret the magnitude of any differences in outcome measures. Effect size values are reported as eta squared and thresholds were set at: *g* < 0.2 trivial effect, g = 0.2 small effect, *g* = 0.5 medium effect, and *g* = 0.8 large effect (Durlak 2009). Statistical power of the study was calculated post hoc using G*Power statistical software (v3.1.9.7, Düsseldorf, Germany) using the effect size, group mean, SD and sample size of the primary outcome measures, these being AMPKα2 and mTOR. Power was calculated as 0.6, as such the data presented here should be interpreted with caution and treated as preliminary data in a cohort of competitive cyclists.

## 4 Results

### 4.1 AMPK Pathway

**FIGURE 2:**
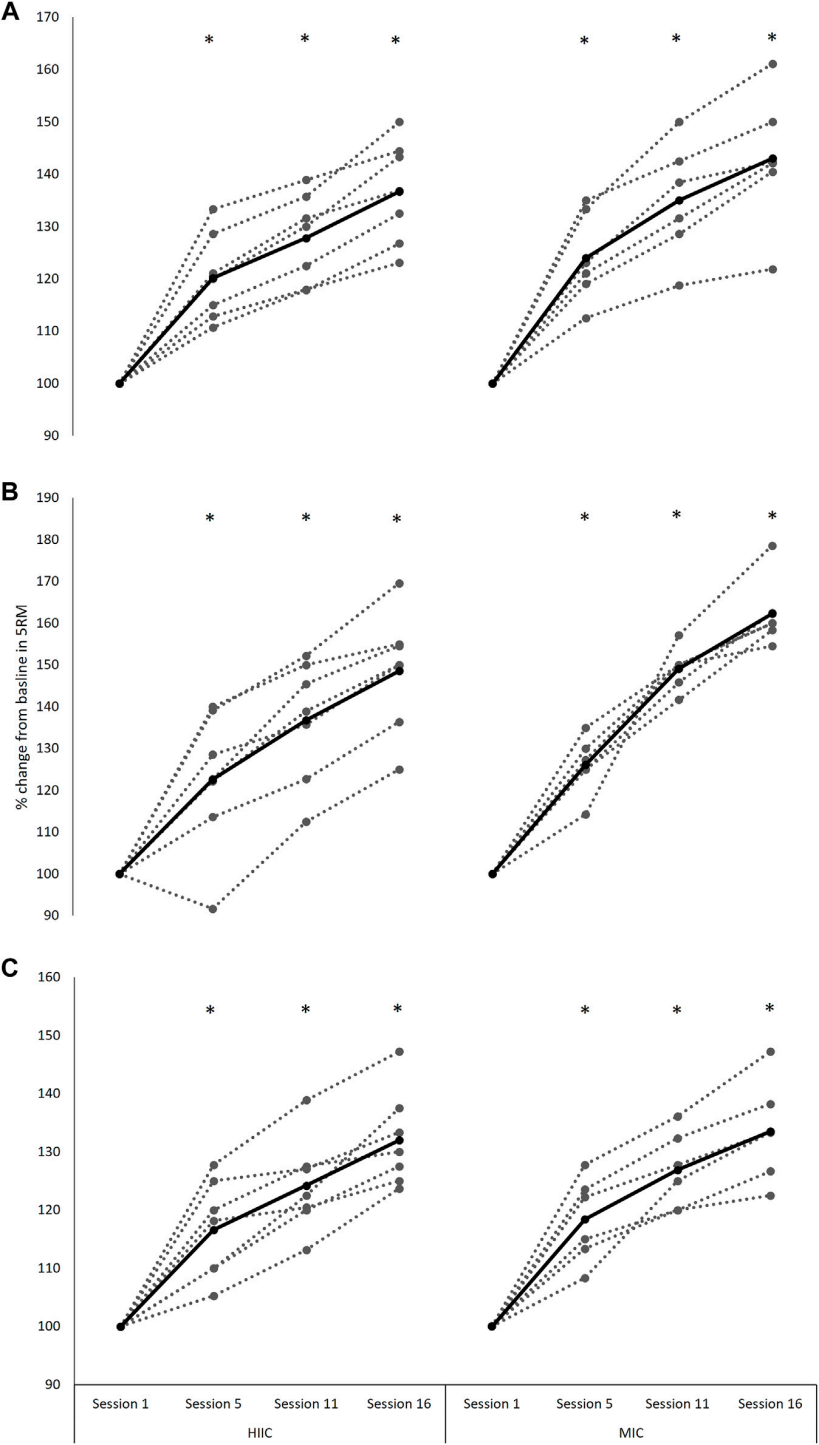
Individual (dashed lines and grey dots) and mean (black lines and black dots) response in phosphorylation of the AMPK signaling pathway in the MICT (*n* = 6) and HIIT (*n* = 7) groups including representative images. Pre = pre-training intervention, post = post-training intervention, RES = resistance exercise; MICT = moderate intensity cycling; HIIT = high intensity interval cycling.

The signaling response of the protein kinases associated with the AMPK pathway are presented in Figure 2 including representative images. There were no interaction effects (AMPKα2 *p =* 0.620; p38α *p* = 0.366; ERK *p =* 0.517; STAT2 *p =* 0.453; HSP27 *p =* 0.456; p53 *p =* 0.959) nor effects of time (AMPKα2 *p =* 0.283; p38α *p =* 0.585; ERK *p =* 0.512; STAT2 *p =* 0.456; HSP27 *p =* 0.927; p53 *p =* 0.092) for the phosphorylation of targets in response to the single concurrent exercise stimulus conducted pre- and post-training intervention.

### 4.2 mTOR Pathway

**FIGURE 3:**
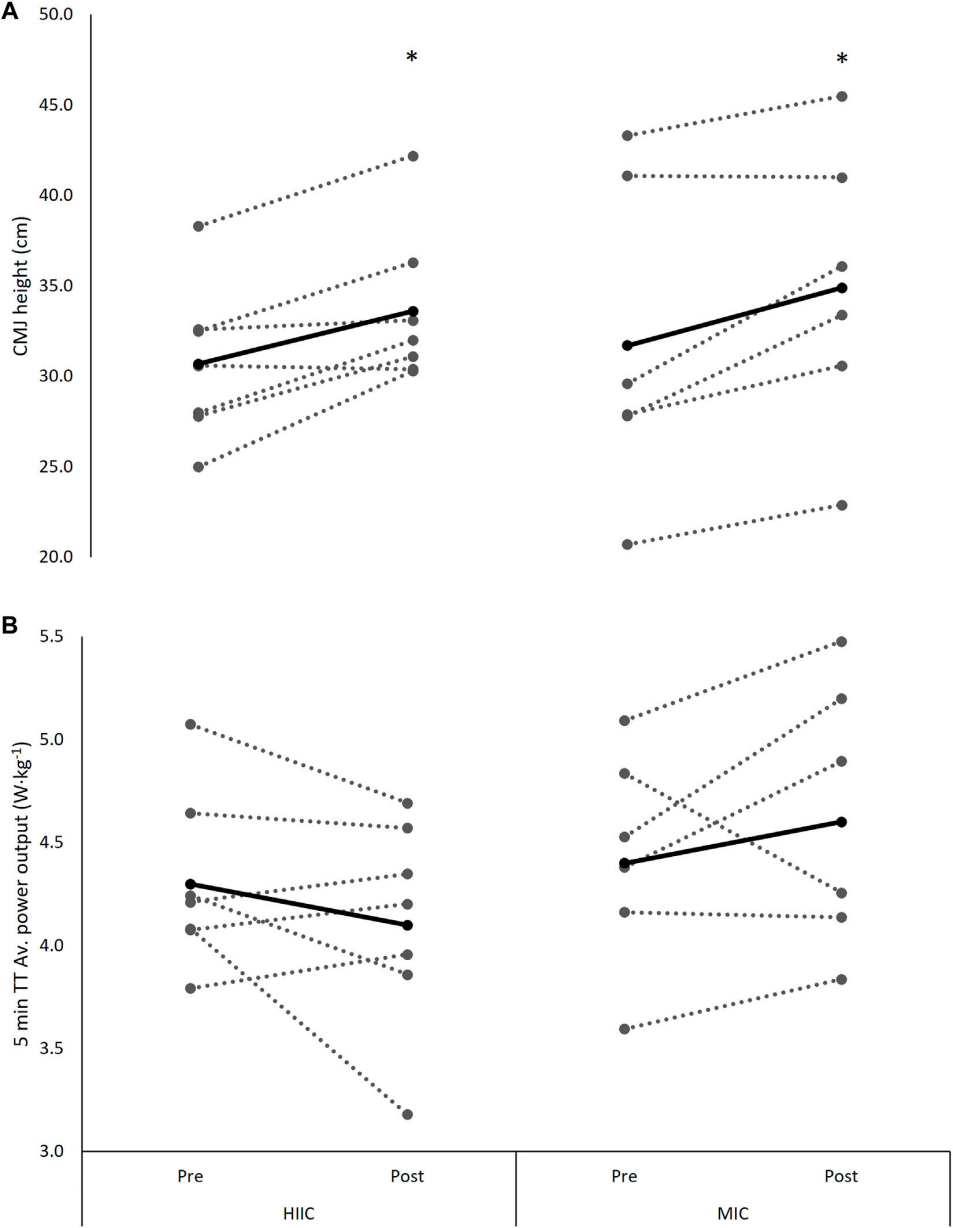
Individual (dashed lines and grey dots) and mean (black lines and black dots) response in phosphorylation of the mTOR signaling pathway in the MICT (*n* = 6) and HIIT (*n* = 7) groups including representative images. Pre = pre-training intervention, post = post-training intervention, RES = resistance exercise; MICT = moderate intensity cycling; HIIT = high intensity interval cycling.

The signaling response of the protein kinases associated with the mTOR pathway are presented in Figure 3 including representative images. There were no interaction effects (Akt *p* = 0.339; mTOR *p* = 0.275; p70S6K *p* = 0.073) nor effects of time (Akt *p* = 0.721; mTOR *p* = 0.473; p70S6K *p* = 0.940) for the phosphorylation of targets during the single concurrent exercise stimulus conducted pre- and post-training intervention.

### 4.3 Training Compliance

Training compliance was high in both groups, with 98.2 ± 3.0% and 98.9 ± 2.6% of total sessions completed throughout the training intervention period for the RES + HIIT and RES + MICT groups, respectively. There was no significant difference in the compliance between the two groups (*p* = 0.356, Hedge’s g = 0.23).

### 4.4 Training Load

Across the intervention HR (% of max HR) was lower during RES + MICT (91.8 ± 4.3%) than the RES + HIIT (96.9 ± 3.1%) condition (*p* = 0.020, Hedge’s g = 1.28). Overall, across the 8-weeks intervention there were no significant differences between RES + MICT and RES + HIIT in total prescribed (*p* = 0.560) and non-prescribed (*p* = 0.200) load, nor prescribed (*p* = 0.746) and non-prescribed (*p* = 0.315) TRIMP (Table 2).

**TABLE 2:** Training load metrics between RES + MICT and RES + HIIT conditions across the training interventions.

	Prescribed (laboratory)			Non-prescribed (non-laboratory)		
	RES + MICT	RES + HIIT	Hedge’s g	RES + MICT	RES + HIIT	Hedge’s g
Load (AU)						
Sum over intervention^*^	3335 ± 941	3665 ± 877	0.37	5124 ± 2247	8419 ± 5039	0.76
Week 1	427 ± 79	480 ± 114	0.49	501 ± 587	1660 ± 1367	0.99
Week 2	360 ± 116	451 ± 174	0.56	580 ± 739	2048 ± 3127	0.89
Week 3	447 ± 273	440 ± 287	0.02	408 ± 307	1201 ± 1005	0.96
Week 4	340 ± 155	246 ± 123	0.63	623 ± 548	1341 ± 827	0.94
Week 5	473 ± 157	571 ± 286	0.39	1073 ± 1171	788 ± 947	0.24
Week 6	491 ± 132	543 ± 342	0.18	1022 ± 907	694 ± 1193	0.29
Week 7	399 ± 322	486 ± 255	0.28	690 ± 885	359 ± 438	0.45
Week 8	399 ± 251	447 ± 242	0.18	227 ± 331	328 ± 566	0.20
TRIMP (AU)						
Sum over intervention^*^	1822 ± 165	1773 ± 305	0.18	2623 ± 1275	4209 ± 3180	0.59
Week 1	232 ± 54	243 ± 33	0.19	296 ± 394	833 ± 604	0.96
Week 2	177 ± 61	216 ± 50	0.66	415 ± 402	724 ± 930	0.39
Week 3	225 ± 69	210 ± 60	0.34	231 ± 153	533 ± 357	0.96
Week 4	214 ± 40	183 ± 87	0.41	431 ± 342	547 ± 480	0.26
Week 5	246 ± 54	206 ± 45	0.72	389 ± 395	693 ± 789	0.45
Week 6	185 ± 54	292 ± 82	1.41	324 ± 223	494 ± 703	0.29
Week 7	323 ± 67	194 ± 112	1.27	275 ± 330	217 ± 262	0.18
Week 8	221 ± 112	229 ± 106	0.07	261 ± 339	168 ± 321	0.26

Note: Values presented as mean ± SD, unless otherwise stated, ^*^mean per participant. AU, arbitrary units; MICT, moderate intensity continuous training; HIIT, high intensity interval training; Hedge’s g = effect size of difference between RES + MICT, and RES + HIIT.

### 4.5 Body Composition

There were no interaction effects, nor effects of time across the parameters of body mass (interaction *p* = 0.956.; time *p* = 0.784), body fat % (interaction *p* = 0.980; time *p* = 0.814), fat-free mass (interaction *p* = 0.919; time *p* = 0.853), sum of 7 (interaction *p* = 0.978; time *p* = 0.811), sum of upper-body (UB) (interaction *p* = 0.828; time *p* = 0.907), and sum of lower-body (LB) (interaction *p* = 0.511; time *p* = 0.416) (Table 3).

**TABLE 3:** Baseline and pre-to post-training change in body composition parameters for the MICT and HIIT groups.

Condition	Body Mass (kg)	Sum of 7 (cm)	Body Fat (%)	Fat-free Mass (kg)	Sum of UB (cm)	Sum of LB (cm)
MICT (baseline)	68.5 ± 8.6	56.6 ± 22.4	10.0 ± 3.9	54.2 ± 6.8	19.7 ± 4.9	17.6 ± 7.7
MICT (change)	0.1 ± 0.2	1.6 ± 5.1	0.3 ± 0.9	0.8 ± 0.6	0.7 ± 1.8	-0.3 ± 1.3
MICT Hedge’s g	0.01	0.06	0.07	0.10	0.13	0.03
HIIT (baseline)	74.7 ± 5.0	64.6 ± 14.9	11.3 ± 2.6	59.6 ± 6.8	22.0 ± 5.1	17.3 ± 4.5
HIIT (change)	-0.3 ± 0.2	2.0 ± 7.4	0.3 ± 1.3	0.2 ± 2.0	-0.2 ± 1.7	-0.4 ± 3.2
HIIT Hedge’s g	0.02	0.12	0.10	0.02	0.03	0.08

Note: Values presented as mean ± SD. MICT, moderate intensity continuous training; HIIT, high intensity interval training; UB, upper body; LB, lower body.

### 4.6 Maximal Strength

**FIGURE 4:**
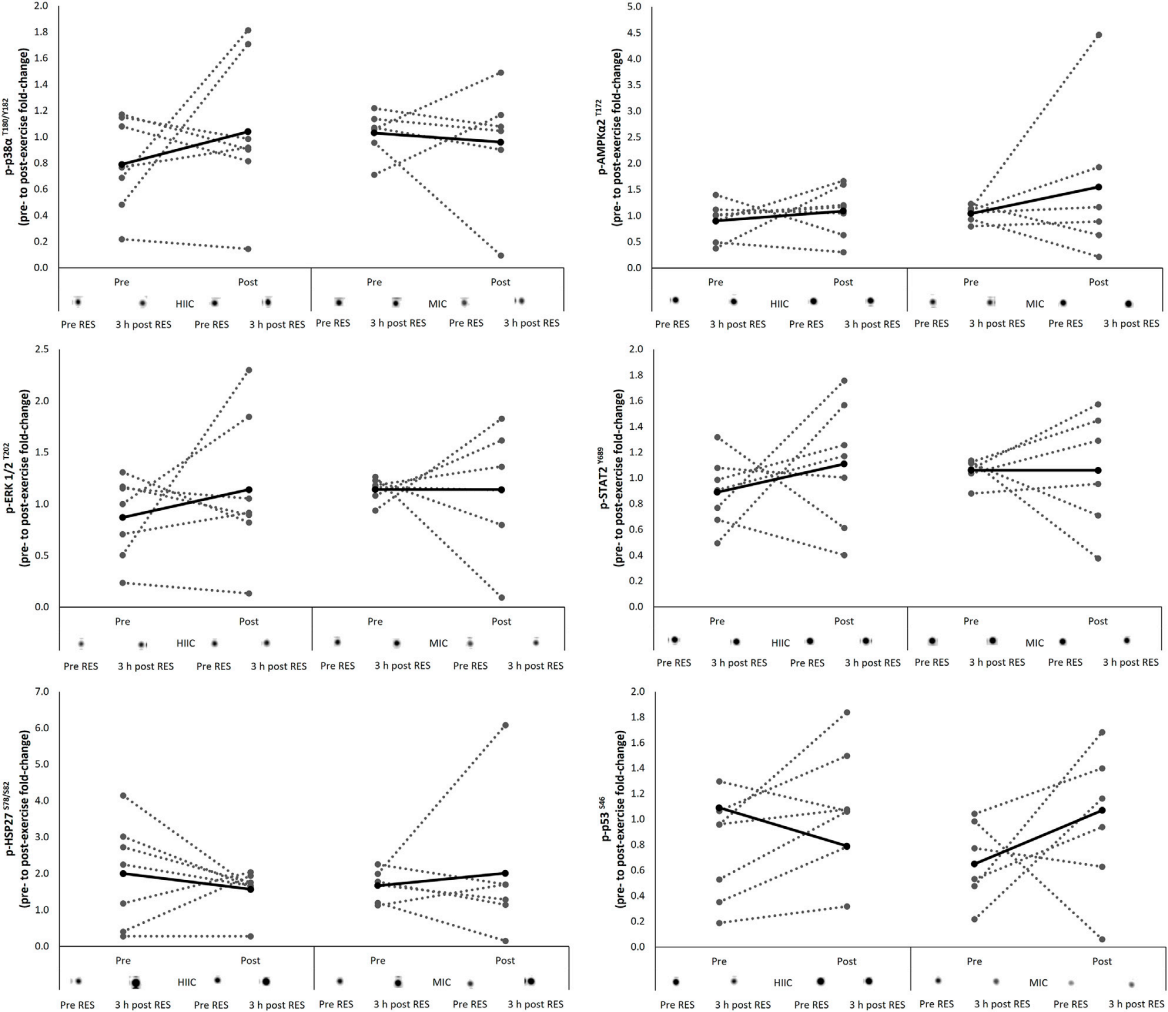
Individual (dashed lines and grey dots) and mean (black lines and black dots) 5-RM **(A)** back-squat **(B)** split-squat performance and **(C)** calf-raise performance (% change from baseline) across the intervention period in the MICT (*n* = 6) and HIIT (*n* = 7) groups. Absolute baseline values for back-squat, split-squat and calf-raise were; 89.2 ± 14.6 and 95.4 ± 22.7 kg, 51.7 ± 9.3 and 56.1 ± 7.9 kg, 83.3 ± 14.0 and 102.1 ± 9.9 kg for MICT and HIIT, respectively. *, significantly different from session 1 (*p* < 0.001). MICT = moderate intensity continuous training; HIIT = high intensity interval training.

A main effect of time was observed for each of the maximal strength exercises; the back-squat (*F*_[1.4,15.1]_ = 130.590, *p* < 0.001, Hedge’s g = 4.65), split-squat (*F*_[2.1,23.3]_ = 137.981, *p* < 0.001, Hedge’s g = 5.88), and calf-raise (*F*_[2.0,21.8]_ = 115.410, *p* < 0.001, Hedge’s g = 5.95), with all improving post interventions (*p* < 0.001) (Figure 4). There were no interaction effects for any of the three exercises (back-squat *p* = 0.331; split-squat *p* = 0.067; calf raise *p* = 0.750).

### 4.7 Cycling Performance

**FIGURE 5:**
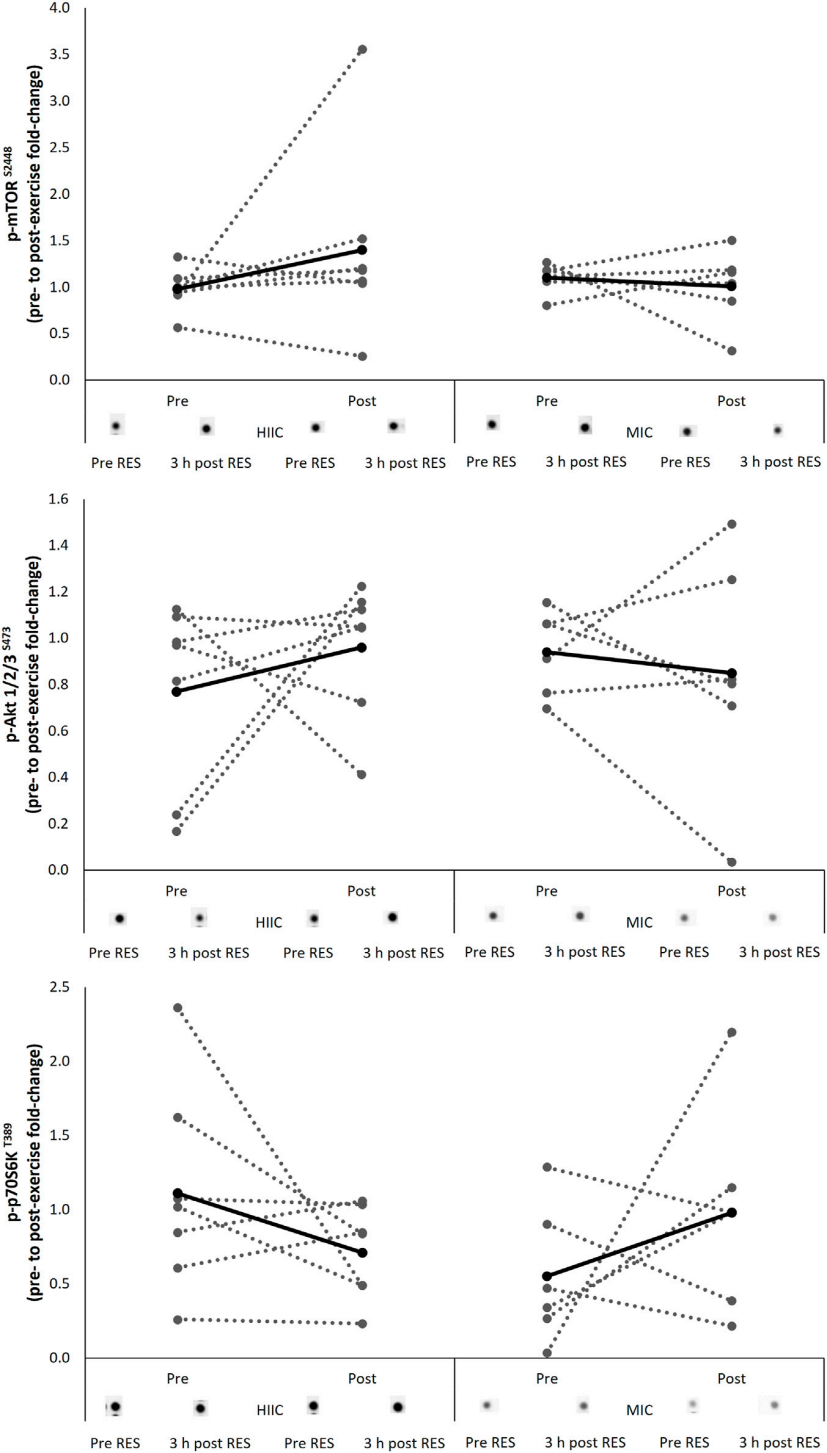
Individual (dashed lines and grey dots) and mean (black lines and black dots) **(A)** CMJ height and **(B)** 5 min TT performance at pre- and post-intervention in the MICT (*n* = 6) and HIIT (*n* = 7) groups. MICT = moderate intensity cycling; HIIT = high intensity interval cycling. *, significantly different from pre-to post-intervention (*p* < 0.05).

There was a time × group interaction for V̇O_2peak_ from pre-to post-training, with the RES + MICT group displaying a preferential response in comparison to that of the RES + HIIT group (*F*_[1,11]_ = 9.649, *p* = 0.010, Hedge’s g = 0.83). There were no significant interaction nor time effects across the measures of power at 2 mmol L^−1^ (interaction *p* = 0.759; time *p* = 0.967) or power at 4 mmol L^−1^ (interaction *p* = 0.738; time *p* = 0.856, Table 4). Similarly, there were no significant interaction (*p* = 0.335) nor time effects (*p* = 0.967) for 5 min TT performances (Figure 5).

**TABLE 4:** Pre to post-training change in aerobic thresholds and V̇O_2peak_ for the MICT and HIIT groups.

Condition	Power at 2 mmol L^−1^ (W)	Power at 4 mmol L^−1^ (W)	V̇O_2peak_ (ml kg^−1^ min^−1^)
MICT (baseline)	210 ± 35	248 ± 30	57.9 ± 7.4
MICT (change)	-4.3 ± 23.3	-1.7 ± 14.1	2.2 ± 2.0
MICT Hedge’s g	0.13	0.07	0.29
HIIT (baseline)	222 ± 29	262 ± 34	54.1 ± 6.7
HIIT (change)	3.3 ± 16.2	5.8 ± 18.7	-2.7 ± 3.4
HIIT Hedge’s g	0.12	0.19	0.43

Note: Values presented as mean ± SD. MICT, moderate intensity continuous training; HIIT, high intensity interval training.

### 4.8 Countermovement Jump Height Performance

There was a main effect of time for the change in CMJ performance across the training programme (*F*_[1,11]_ = 7.849, *p* = 0.017, Hedge’s g = 0.51), with no interaction effect observed (*p* = 0.963, Figure 3).

## 5 Discussion

This study aimed to determine whether the acute molecular response to concurrent exercise stimuli is affected by training status i.e., pre *vs* post training, or differentially affected relative to the intensity of the endurance training prescribed. Further, whether the intensity of endurance stimuli throughout a concurrent training block would affect performance outcomes. These questions were examined in the context of an endurance trained, but strength training naïve cohort. The major findings were that 1) the mean acute molecular response was comparable before and after the training intervention and not differentially activated by the intensity of endurance stimuli. 2) the intensity of endurance stimuli had no effect on performance outcomes, despite the interventions improving strength and power parameters; At this point it should be noted that these findings were observed in a relatively small cohort of well-trained endurance cyclists and as depicted in Figures 2, 3, there was considerable variability in the individual molecular responses to the concurrent exercise stimuli. Whilst standardized effect sizes have been employed assist with to interpretation of any significant effects, the data presented here should be interpreted with caution and treated as preliminary data due to a low n and statistical power. It was, of course, very challenging to recruit competitive cyclists who were willing to have their training modified for an 8-week period and undergo muscle tissue sampling.

Given the nature of the interference effect, the observation of strength outcomes is pertinent in research aiming to optimize concurrent training methods. Silva et al. (Silva et al., 2012) reported no group differences in knee extension and leg press performance, with an average change across groups of 33 and 42%, respectively. These observations were specific to an untrained female cohort which had been assigned to 11 weeks of concurrent training with either a continuous or high intensity endurance component. Fyfe et al. (Fyfe et al., 2016b) reported slightly smaller improvements in maximal leg press strength, with a 29 and 28% change in the high and moderate-intensity conditions, respectively. These are largely consistent with the findings of this study, such that significant improvements in lower-body maximal strength were observed, with no effects relating to the endurance intensity of the concurrent stimulus. Specifically, this work observed average performance improvements of 39, 55, and 33% in the back-squat, split-squat, and calf-raise, respectively.

The improvements in strength were not reported in conjunction with significant improvements in fat free mass or a reduction in the sum of lower-body sites. These parameters were used as a rudimentary assessment of hypertrophy. The expectation is that 8 weeks of resistance training in strength naïve individuals would likely result in improvements in strength and a surrogate assessment of hypertrophy. Therefore, these data are suggestive of neuromuscular adaptations explaining the enhanced strength performance in the respective exercises. Other research has demonstrated hypertrophy because of resistance training across a similar timeframe (Ogasawara et al., 2014; Boone et al., 2015). However, this observation of hypertrophy might be explained by such work incorporating more sophisticated techniques, such as magnetic resonance imaging (MRI), X-ray computerized tomography, or ultrasound.

Consistent with the response in parameters of strength, the current study observed an improvement in CMJ performance across the training intervention, with no group effects. This contrasts with the work of Fyfe et al. (Fyfe et al., 2016b), which reported no improvement for peak CMJ height amongst the two concurrent training groups. Interestingly, these authors assessed numerous aspects of CMJ performance, with the only significant improvement reported for peak velocity in the moderate-intensity group. Power is a critical parameter in the context of concurrent training, as it is the only outcome to detrimentally change relative to resistance training in isolation, according to a recent meta-analysis (Schumann et al., 2021). Whilst it is positive that both concurrent training programmes from this study resulted in improved CMJ performance, it is not possible to place this finding in the context of the interference effect, given the design used in this work.

Whilst power output at blood lactate concentrations of 2 and 4 mmol L^−1^ were not differently affected by concurrent training with MICT or HIIT, post intervention the RES + MICT condition resulted in preferential changes in V̇O_2peak_ when compared with RES + HIIT. This is in contrast with previous similar research which reported peak aerobic power responded preferentially to a higher-intensity endurance stimulus (Fyfe et al., 2016b). Here, somewhat surprising preferential effect of RES + MICT on V̇O_2peak_ is difficult to explain, although this preferential effect may be related to variances in non-prescribed load and TRIMP between RES + MICT and RES + HIIT. Whilst no significant difference in non-prescribed load and TRIMP were observed between RES + MICT and RES + HIIT, medium effect sizes were present and indicated that RES + HIIT constituted greater non prescribed load and TRIMP than RES + MICT. Although is it perhaps more logical that the greater training load elicited by RES + HIIT would result in greater improvements in V̇O_2peak_, it is also possible that the greater training load resulted in participants being more fatigued at the time of the post-intervention aerobic assessments, although this remains speculative. Others have reported a reduction in endurance performance following an investigation into the manipulation of endurance intensity with concurrent training (Silva et al., 2012). However, these authors used a particularly poor marker of endurance performance; the maximum number of repetitions achieved at 70% 1RM. While such methods undoubtedly characterize the fatigue response of local musculature or endurance capacity, they are reported to be a poor marker of applied endurance performance (Currell and Jeukendrup 2008). This work sought to improve ecological validity and employed a TT effort. Although the cyclists achieved improvements in strength and power (as assessed by 1RMs and CMJ), TT performance was unchanged following both RES + MICT and RES + HIIT interventions. This is perhaps unsurprising as whilst participants were naïve to strength training, they were well trained endurance cyclists, and regularly competed in events including time trials. As such, it is possible that the training status and experience of the participants prevented the transfer of improvements strength and power to improved TT performance.

While this study did not attempt to examine the role of exercise intensity in the context of an interference effect i.e., a concurrent stimulus *vs* resistance only, it did examine whether endurance exercise intensity can be modified to improve a concurrent stimulus. It was important to address this question across the course of a short-term training programme. The seminal work in the field and first to examine the challenges of concurrent programming, was conducted across a short-term training intervention (Hickson 1980). Given that the divergence in response between groups occurred from the 5–10 weeks, it would seem appropriate to address such questions over at least a similar timeframe. Indeed, this consideration has been raised previously (Hamilton and Philp 2013), with authors stressing the requirement for research observing the molecular responses across a longer timeframe than the popular model of acute observations.

Previous efforts to examine molecular responses to concurrent stimuli across a period of greater than 5 weeks are limited. de Souza et al. (de Souza et al., 2012) reported total p70S6K and phosphorylated Akt protein expression to increase from pre-to post-training time points. Fyfe et al. (Fyfe and Loenneke 2018) observed greater basal phosphorylation of p70S6K, with both mTOR and rpS6 phosphorylation still increasing in response to concurrent exercise following 8 weeks of training. Conversely, Kazior et al. (Kazior et al., 2016) reported a reduction in total p70S6K content, but in combination with an increase in mTOR and Akt protein expression post-intervention. The literature has characterized the responses amongst recreationally active individuals, with none of the methods specifically comparing the acute response to concurrent exercise before and after a training intervention. Arguably, a design of this nature would better support conclusions regarding the role of training status in the molecular response to acute concurrent exercise. The importance of training status and its ability to modulate both the specificity and magnitude of training adaptations has previously been described in the literature (Fyfe and Loenneke 2018).

Fernandez-Gonzalo, Lundberg, and Tesch (Fernandez-Gonzalo et al., 2013) utilized an, arguably, improved design and assessed acute molecular responses to a concurrent stimulus in both the pre- and post-training condition. While the activation status of mTOR, rpS6 and eEF2 remained unaltered, p70S6K phosphorylation increased in the trained state. This would counter the hypothesis of an attenuation in, or a more mode-specific response to, exercise in the trained state. However, these findings were in the context of 5 weeks of training amongst moderately active individuals, and therefore not reflective of a prolonged training history. The major finding from the present study was a lack of a time effect in protein phosphorylation fold-change from pre-to post-intervention. This consistency in early exercise response before and after the training intervention is suggestive of either 1) continued adaptation after 8 weeks of training, or 2) a poor exercise stimulus from the onset of the intervention. The former seems more likely in this scenario given the improvement in strength and power parameters.

Previous literature concerning the role of endurance exercise intensity during concurrent training has employed an endurance followed by resistance exercise order for the concurrent training stimulus (Silva et al., 2012; Fyfe et al., 2016b). Employing this exercise sequence might stress the neuromuscular element of residual fatigue within a concurrent training paradigm (Lepers et al., 2000). However, a meta-analysis has indicated a beneficial effect of a resistance followed by endurance exercise order for lower-body strength adaptation across a short-term concurrent training programme (Eddens et al., 2018). This would suggest that such an exercise sequence provides an appropriate model to examine the optimization of concurrent training methods. It is the development of strength, which is potentially inhibited with this training paradigm, and as such, the methods should strive to elicit adaptation in strength parameters. This would constitute a more ecologically valid paradigm to investigate the role of endurance exercise intensity and is the model adopted here.

This work does not support the idea of endurance exercise intensity negatively modulating the adaptive response of resistance exercise structured in a short-term concurrent training paradigm. This agrees with previous work in untrained cohorts (Silva et al., 2012; Fyfe et al., 2016b) and could support the concept of volume or frequency of endurance stimuli proving a more potent mediator of adaptation to concurrent training (Jones et al., 2016). While the design of the work does not confirm whether either endurance training condition had an inhibitory effect on strength adaptation, the magnitude of strength adaptation observed is similar compared with that reported following short-term resistance training in strength naïve individuals (Del Balso and Cafarelli 2007). Further, the complexities of research design in concurrent training literature should also be considered. There are many acute training variables encountered when implementing a concurrent training paradigm, such as intensity, volume, sequence, relief period, and frequency. While this study manipulated the variable of intensity and attempted to control for other components, it is not possible to identify the effect of employing alternate conditions with regards to these variables, and the resultant outcome on performance. It should also be acknowledged that while the group difference in non-prescribed endurance training load did not reach statistical significance nor a large effect size, it could be physiologically relevant. Furthermore, while ensuring control by delivering comparable work-matched endurance stimuli, the ecological validity of work-matched endurance interventions in trained cohorts has been questioned (Seiler and Tønnessen 2009). This work provides valuable information regarding the response to HIIT at 85% V̇O_2peak_, which represents a training stimulus that athletes might undertake, however caution should be exercised in extrapolating these findings to interval training of higher intensities, such as V̇O_2max_.

It was confirmed that the intensity of endurance exercise (as part of a concurrent training stimulus) had no effect on performance outcomes, following short-term concurrent training. Importantly, this was in the context of improvements in strength and power parameters. Further, the acute molecular response to a concurrent exercise stimulus was comparable before and after the training intervention, suggesting that training status had no effect on the molecular responses assessed. Finally, the molecular responses to a concurrent exercise stimulus were not differentially activated by the intensity of endurance stimuli. These findings add further support to the growing argument that any interference effects in a concurrent training paradigm are not mediated by the mTOR-AMPK axis. However, as previously acknowledged, due the relatively low sample size, parallel groups design and large inter individual variability within the molecular data these inferences should be interpreted with caution and treated as preliminary data.

## Data Availability

The raw data supporting the conclusions of this article will be made available by the authors, without undue reservation.
